# Artificial Intelligence Algorithm-Based Computed Tomography Image in Assessment of Acute Renal Insufficiency of Patients Undergoing Percutaneous Coronary Intervention

**DOI:** 10.1155/2022/2214583

**Published:** 2022-02-28

**Authors:** Xiuming Zhang, Tao Liu, Chunhua Tian

**Affiliations:** Department of Nephrology, Inner Mongolia Baogang Hospital, Baotou 014010, Inner Mongolia Autonomous Region, China

## Abstract

This study was aimed to analyze the changes in renal function of patients undergoing percutaneous coronary intervention (PCI) surgery and the characteristics of their computed tomography (CT) image based on artificial intelligence algorithms. In this study, 104 patients with coronary atherosclerotic heart disease (CAHD) were treated as the research objects. They were divided into an experimental group (patients who underwent CAG and PCI within 1 week after enhanced coronary CT (ECCT)) and the control group (patients who underwent CAG and PCI within 1–3 weeks after ECCT). Renal imaging scans of patients were performed by CT based on discrete inseparable shear transform (DNST) optimized algorithm, which was named as O-DNST. The results showed that the serum creatinine (Scr), blood urea nitrogen (BUN), and urine protein (UP) levels of patients in the experimental group were significantly higher than those of the control group 24–72 hours after surgery, while the levels of endogenous creatinine clearance (Ccr) and estimated glomerular filtration rate (eGFR) were significantly lower than those of the control group (*P* < 0.05). The levels of *β*2 microglobulin (*β*2-MG), C-reactive protein (CRP), interleukin 6 (IL-6), and tumor necrosis factor (TNF-*α*) in the experimental group were significantly higher than those in the control group 24–72 hours after surgery (*P* < 0.05). The incidence of contrast-induced nephropathy (CIN) in the experimental group (15.38%) was significantly higher than that in the control group (5.8%), and the difference was statistically significant (*P* < 0.05). The results showed that repeated application of contrast agent in a short period of time can promote the increase of serum inflammation levels in PCI patients, which may be a risk factor for CIN in PCI patients.

## 1. Introduction

Coronary atherosclerotic heart disease (CAHD) is caused by atherosclerotic lesions in coronary arteries that cause stenosis or obstruction of the vascular lumen, resulting in myocardial ischemia, hypoxia, or necrosis, so it is often referred to as “coronary heart disease (CHD)” [1]. CHD is a common disease of middle-aged and elderly people, frequently occurring, and seriously endangering people's lives. Most people do not have any symptoms at ordinary times, work, study, and life are as usual, but they often have signs of myocardial ischemia, such as feeling frontal discomfort or symptoms of fatigue. Although the symptoms are mild, if the electrocardiogram is performed in time, myocardial ischemia will be found [2–4]. The treatment of CHD mainly includes drug therapy, interventional therapy, and surgical coronary artery bypass graft. Medical treatment is the cornerstone of CHD treatment, regardless of the degree of coronary artery stenosis or whether the patient has a stent or a heart bypass. Once diagnosed with CHD, patients need long-term adherence to oral prognostic drugs, but they can only relieve symptoms, stabilize plaques, and prevent acute myocardial infarction [5, 6]. Coronary artery bypass grafting refers to the use of the patient's own great saphenous vein or other arteries to connect the distal end of the narrowed coronary artery with the aorta to supply blood to the distal end of the myocardium to improve the symptoms of angina pectoris and improve life treatment, but the operation is complicated and risky [7]. Percutaneous coronary intervention (PCI) has the advantages of simple operation, small trauma area, and quick postoperative recovery. It can quickly rebuild coronary blood vessels in emergency situations, which can effectively alleviate the symptoms of patients and improve the quality of life [8].

With the extensive clinical application of contrast technology, especially in elderly patients with severe comorbidities, contrast-induced nephropathy (CIN) has become the third leading cause of hospital-acquired renal failure, accounting for the largest incidence of 11% [9, 10]. In many studies, CIN is defined as acute renal function decline that occurs after intravascular contrast media is used for no other reason. The specific manifestations are summarized as follows: 48–72 hours after using the contrast agent, the serum creatinine level rises by 44 *μ*mol/L, or 25% higher than the baseline serum creatinine level [11]. As for the pathogenesis of CIN, it is still unclear. Some studies believe that renal blood flow reduction, especially renal medulla ischemia, direct cytotoxicity, and the osmotic pressure and viscosity of the contrast agent play an important role in the pathogenesis of CIN [12–14]. At present, it is believed that the original renal insufficiency is the most important risk factor for the occurrence of CIN. There is no evidence-based medicine to prove that a certain drug has a definite preventive effect on CIN. However, some studies have confirmed that certain drugs can play a role in reducing the incidence of CIN, such as adenosine antagonists, statins, vitamin C, and calcium channel blockers. Other preventive measures include: maintaining water and electrolyte balance, closely monitoring renal function indicators before and after angiography, actively dealing with complications, and strengthening nutritional support, which show differences in effect, so the focus of treatment of CIN is to prevent [15]. In recent years, CT enhancement has been widely used in clinical heart and coronary artery imaging and has become an important noninvasive imaging method for the diagnosis and detection of CHD. Water-soluble iodine contrast agents are commonly used in CT examinations, and the adverse reactions caused by iodine-containing contrast agents, especially contrast agent nephropathy, have attracted the attention and attention of clinicians and radiologists [16]. How to prevent contrast nephropathy and life-threatening reactions is an urgent issue that deserves attention and must be properly handled.

Intelligent medical imaging uses artificial intelligence technology to analyze and process the scanned images of commonly used medical imaging technologies such as X-ray, CT, magnetic resonance imaging (MRI), and ultrasound and provide diagnostic assistance and prompts [17]. Based on this, 104 patients with CAHD who underwent coronary angiography (CAG) and PCI surgery after the enhanced coronary CT (ECCT) were treated as the research objects. They were divided into an experimental group (patients who underwent CAG and PCI within 1 week after ECCT) and the control group (patients who underwent CAG and PCI within 1–3 weeks after ECCT). The preoperative and postoperative renal function indicators, inflammatory indicators, and the incidence of CIN in the two groups were compared to deeply analyze the clinicopathological characteristics of acute renal function injury caused by the application of contrast agents in PCI surgery, aiming to provide reference for selection of clinical prevention and treatment strategies for CIN.

## 2. Materials and Methods

### 2.1. Research Objects

In this study, 104 patients with CAHD who underwent CAG and PCI surgery in hospital from March 2018 to May 2021 were selected as the research subjects after coronary CT examination with enhanced coronary artery. There were 58 males and 46 females, with an age range of 20–72 years. This study had been approved by the ethics committee of the hospital, and the patients and their family members had understood the research situation and signed the informed consent forms.

Inclusion criteria were defined as follows: patients who signed the informed consents; patients with angina or severe coronary artery stenosis; patients who did not receive medication or surgical treatment; and patients older than 18 years old.

Exclusion criteria were given as follows: patients over 72 years old; patients with hyperthyroidism; patients who had received interventional therapy; patients who were allergic to iodine contrast agents; patients with severe renal dysfunction; patients recently taking a large number of nephrotoxic drugs; and patients who were suspected with severe left main stem disease.

### 2.2. Grouping

The included patients were randomly divided into an experimental group (52 cases) and a control group (52 cases). Patients in the experimental group underwent CAG and PCI surgery within 1 week after ECCT; whereas those in the control group underwent CAG and PCI surgery within 1–3 weeks after ECCT.

### 2.3. CT Examination

CT Scanner was adopted in this study. The patient was required to keep a supine position and scan from the diaphragm to the symphysis pubis. Scanning parameters were given as follows: tube voltage was 120 kV, tube current was 220 mA, matrix was 256 × 256, conventional scanning layer thickness was 5 mm, and layer spacing was 5 mm. The contrast agent iohexol was injected from the cubital vein at a flow rate of 2.5 mL/s, the scanning time of the arterial phase was 30 seconds, the scanning time of the parenchymal phase was 50 seconds, the scanning time of the delayed phase was 100 seconds, the reconstruction layer thickness was 1.5 mm, and the layer spacing was 1.5 mm.

### 2.4. Improved Image Denoising Algorithm Based on DNST (O-DNST)

Shear wave transform [18] is a new type of multiscale geometric analysis method, which is constructed by affine transformations such as scaling, shearing, and translation of the basic function, which embodies the geometric and mathematical characteristics of the function. In this study, the discrete nonseparable shear wave transform (DNST) was introduced as the core algorithm, and the inseparable shear wave generator can be expressed as follows:

(1) $$\begin{array}{c} \Phi_{\text{no}}\left(\eta \right)=U\left(\frac{\eta_{1}}{2},\eta_{2}\right)\Phi \left(\eta \right),\\ \inf_{\eta \in \Gamma }\left|U\left(\eta \right)\right|\geq a_{1},\\ \Gamma =\left\{\eta \in {\left[-\frac{1}{2},\frac{1}{2}\right]}^{2}:\frac{1}{4}\leq \left|\eta_{1}\right|\leq \frac{1}{2},\frac{\eta_{2}}{\eta_{1}}\leq 1\right\}. \end{array}$$

In the above three equations, *U* represented a two-dimensional sector filter, *a*_1_〉0 was a constant, and Φ_no_ represents an inseparable shear wave generator. The shear wave generated by the inseparable shear wave generator can be expressed as follows:

(2) $$\begin{array}{c} \Phi_{\text{no}}{\;}_{\left(i,j,n\right)}\left(x\right){=2}^{\frac{3}{4}i}\Phi_{\text{no}}B_{j}C_{2}x-W_{d1}n. \end{array}$$

(3) $$\begin{array}{c} W_{d1}=\text{diag}\left(d_{1}^{i},d_{2}^{i}\right). \end{array}$$

In the (2) and (3), *W*_*d*1_ represents the sampling matrix, and *d*_1_ and *d*_2_ were the sampling constant for conversion.

The Bayesian algorithm based on the coefficient statistical model in the transform domain has always been a hot topic of image denoising. Constructing a prior probability distribution model for the coefficient edges makes the algorithm have the advantages of better balance of noise suppression and preservation of image details. The NIG distribution was introduced to determine the marginal statistical distribution of DNST coefficients.

It was assumed that the noise image was *Q* and the original image was *P*, then, the following equation could be obtained:

(4) $$\begin{array}{c} Q=P+Z. \end{array}$$

In the above equation, *Z* represented Gaussian noise. After the DNST was introduced, the followingequation could be obtained:

(5) $$\begin{array}{c} q=p+z. \end{array}$$

In equation (5), *q* represented the shear wave coefficient of the noise image through DNST change, *p* represented the shear wave coefficient of the noise-free image, and *z* represented the noise coefficient of the noise-free image. The Bayesian maximum posterior estimation lesson can be expressed as follows:

(6) $$\begin{array}{c} p*\left(q\right)=arg\underset{p}{\max}\left\{h_{p|q}\left(p|q\right)\right\}. \end{array}$$

In equation (6), *h*_*p|q*_(*p|q*) represents the conditional density of observation of *q* versus *p*. Then, the Bayesian was adopted to simplify the processing:

(7) $$\begin{array}{c} p*\left(q\right)=arg\underset{p}{\max}\left\{h_{n}\left(q-p\right)•h_{p}\left(p\right)\right\}. \end{array}$$

(8) $$\begin{array}{c} h_{n}\left(n\right)=\frac{\exp\langle -n^{2}/2\varphi_{n}{\;}^{2}\rangle }{\sqrt{2\pi }\varphi_{n}}. \end{array}$$

In the abovementioned equations, *h*_*n*_(*·*) represented the probability distribution of the noise coefficient, *h*_*p*_(*·*) referred to the prior distribution of the noise-free image coefficient, and *φ*_*n*_ ^2^ represented the Gaussian variance. After equation (8) was substituted into equation (7),

(9) $$\begin{array}{c} p*\left(q\right)=arg\underset{p}{\max}\left[-\frac{{\left(q-p\right)}^{2}}{2\varphi_{n}{\;}^{2}}+\lambda \left(p\right)\right]. \end{array}$$

(10) $$\begin{array}{c} \lambda \left(p\right)=\ln\;h_{p}\left(p\right). \end{array}$$

In equations (9) and (10), *λ*(*p*) was a convex differentiable function. −(*q* − *p*)^2^/2*φ*_*n*_ ^2^+*λ*(*p*) was set as the first derivative, and then *p∗*(*q*) can be calculated to obtain the maximum posterior estimate:

(11) $$\begin{array}{c} \frac{q-p^{*}}{\varphi_{n}{\;}^{2}}+\lambda^{\prime }\left(p^{*}\right)=0. \end{array}$$

Next, an optimal linear interpolation shrink (OLIS) algorithm was proposed to ensure a good threshold effect, which could be expressed as follows:

(12) $$\begin{array}{c} \varphi_{\alpha }^{\text{OLIS}}=\left\{\begin{array}{cc} q-\tau \left(q-\beta \right) & \left|q\right|\leq K\\ 0 & \left|q\right|>K \end{array}\right). \end{array}$$

(13) $$\begin{array}{c} \tau =\frac{\chi_{n}^{2}}{\chi_{q}^{2}}. \end{array}$$

In equations (12) and (13) above, *ꞵ* represented the average value of the corresponding sub-band coefficients, *K* represented the calculated threshold, and *χ*^2^ represented the noise variance.

**Figure 1:**
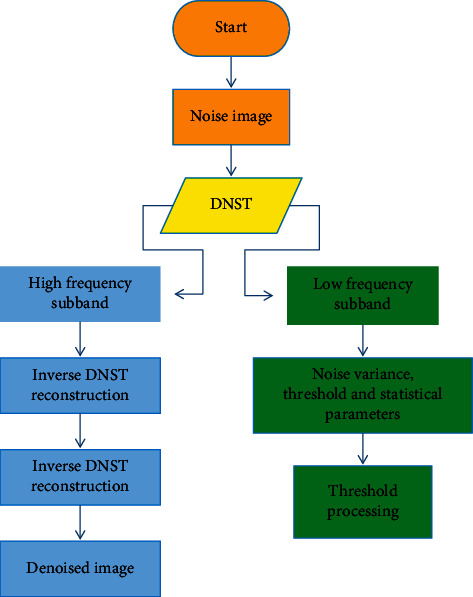
Improved image denoising algorithm process based on DNST.

The above showed an improved image denoising algorithm based on DNST, which was named as O-DNST. In summary, the O-DNST algorithm flow can be shown in Figure 1. Firstly, the discrete inseparable shear waves were adopted to divide the image into multiple high-frequency sub-bands and obtain the corresponding shear wave coefficients and a low-frequency sub-band. Secondly, the noise variance, threshold, and statistical parameters of the high-frequency sub-band were calculated. Thirdly, it could perform threshold processing on all high-frequency sub-band coefficients and perform mixed low-frequency denoising processing on the low-frequency sub-band. Finally, the inverse DNST reconstruction was performed on the low-frequency sub-band to obtain a denoised image.

### 2.5. Indicators of Algorithm Processing Effect

The root mean square error (RMSE) and running time were used as the performance indicators of the algorithm to process CT images.

It was supposed that the width of the image was *L* and the height was *H*, then,

(14) $$\begin{array}{c} RMSE=\sqrt{\frac{\sum_{i=1}^{H}\sum_{j=1}^{L}{\left(v_{ij}-V\right)}^{2}}{L•H}}. \end{array}$$

In equation (14), *v*_*ij*_ represented the gray value of the image, and *V* represented the average value of the gray value of the image.

Discrete separable shear wave transform (DSST) [19], wavelet transform (WT) [20], and DNST were used as controls to compare with the O-DNST algorithm.

### 2.6. Observation Indicators

The general information (gender, age, height, weight, diabetes, hypertension, hyperlipidemia, mild anemia, contrast dose, and Mehran score) of patients were recorded. The preoperative and postoperative (24, 48, and 72 hours) renal function indicators (serum creatinine (Scr), blood urea nitrogen (BUN), endogenous creatinine clearance (Ccr), estimated glomerular filtration rate (eGFR)), urine protein (UP), and inflammatory factors (*β*2 microglobulin (*β*2-MG), C-reactive protein (CRP), interleukin 6 (IL-6), and tumor necrosis factor-*α* (TNF-*α*)) of patients were recorded and compared.

### 2.7. Statistical Methods

The data processing of this study was analyzed by using the SPSS19.0 version statistical software, the measurement data was expressed by the mean ± standard deviation (‾*x* ± *s*), and the count data was expressed by the percentage (%). One-way analysis of variance was used for pairwise comparison. The difference was statistically significant at *P* < 0.05.

## 3. Results

### 3.1. Basic Data of Patients

**Figure 2:**
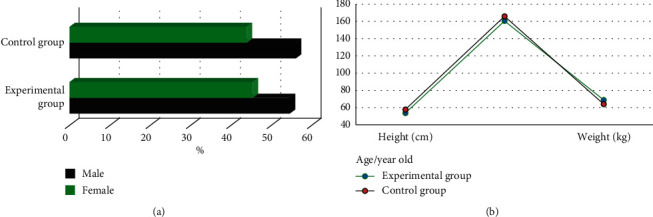
Comparison of gender, age, height, and weight of the two groups of patients. (a) compared the ratio of men to women; and (b) compared the age, height, and weight.

**Figure 3:**
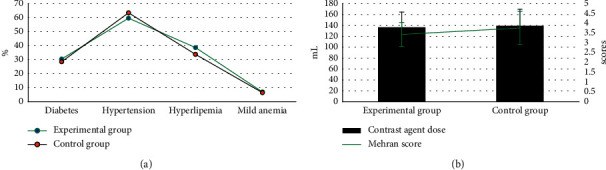
Comparison of diabetes, hypertension, hyperlipidemia, mild anemia, contrast agent dosage, and Mehran score between the two groups. (a) showed the comparison on diabetes, hypertension, hyperlipidemia, and mild anemia; and (b) illustrated the comparison on contrast agent dose and Mehran score.

The two groups of patients were compared in terms of basic information (gender, age, height, weight, diabetes, hypertension, hyperlipidemia, mild anemia, contrast agent dose, and Mehran score). As shown in Figures 2 and 3, the differences were all not statistically significant (*P* > 0.05).

**Figure 4:**
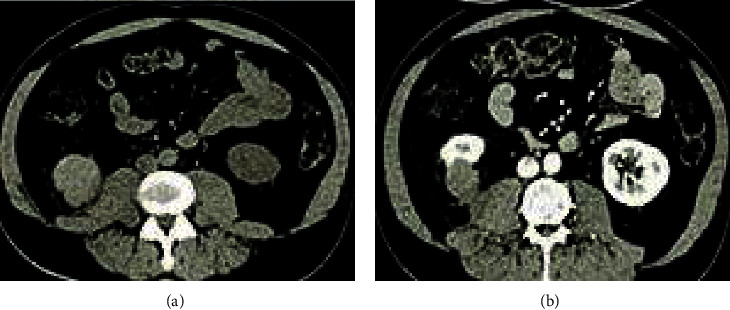
CT image of a patient's kidney (male, 51 years old). The left image was the CT plain scan result, and the right image was the CT enhanced result.

**Figure 5:**
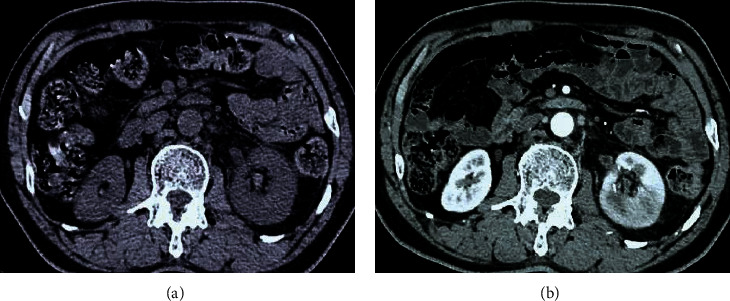
CT image of a patient's kidney (female, 50 years old). The left image was the CT plain scan result, and the right image was the CT enhanced result.

Figure 4 was a CT image of a male patient (51 years old). The kidneys were enlarged on plain scan, and multiple wedge-shaped low-density areas appeared on enhanced scan.  Figure 5 showed a CT image of a female patient (50 years old). The plain scan showed that the kidney is enlarged and the contour was irregular; and the enhanced scan showed the enhancement of the abscess wall, stones, and perinephric involvement.

### 3.2. Performance Analysis of O-DNST Algorithm

**Figure 6:**
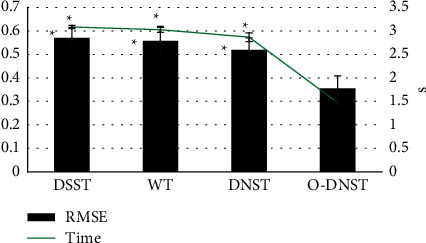
Comparison of image quality evaluation indicators of DSST, WT, DNST, and O-DNST algorithms. ^*∗*^Significant difference compared with the O-DNST algorithm (*P* < 0.05).

Figure 6 showed the comparison of image quality evaluation indicators of DSST, WT, DNST, and O-DNST algorithms. It illustrated that the RMSE and running time of the O-DNST algorithm were significantly lower than the DSST, WT, and DNST algorithms, and the differences were statistically significant (*P* < 0.05).

**Figure 7:**
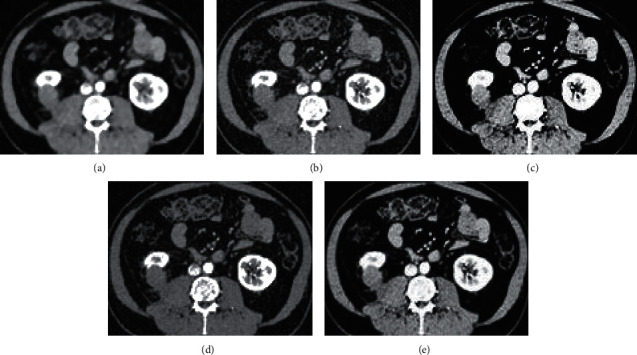
Comparison of image processing effects of DSST, WT, DNST, and O-DNST algorithms. (a) showed the image of CT plain scan; and (b–e) were images processed by DSST, WT, DNST, and O-DNST algorithms, respectively.

As shown in Figure 7 below, the images processed by the DSST, WT, DNST, and O-DNST algorithms showed a certain improvement compared with the original CT images. Among them, the resolution of image processed by WT algorithm was still low, and that of the DSST and DNST algorithms was higher, but there were artifacts, resulting in the subtle display of the organization was not clear. In addition, the O-DNST algorithm showed the best image clarity and tissue resolution, so the overall quality was better than other algorithms.

### 3.3. Comparison of Renal Function Indicators between Two Groups of Patients

**Figure 8:**
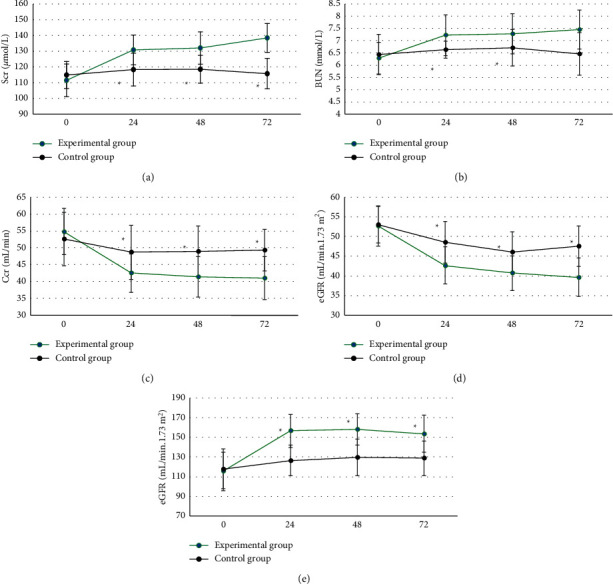
Comparison of renal function indicators between the two groups of patients (0–72 referred to “before surgery”, 24 hours, 48 hours, and 72 hours after surgery, respectively). A ∼ E showed the comparisons of Scr, BUN, Ccr, eGFR, and UP, respectively. ^*∗*^Significant difference compared with the experimental group (*P* < 0.05).

As shown in Figure 8, the preoperative Scr, BUN, Ccr, eGFR, and UP levels of patients in the experimental group and the control group were not statistically different (*P* > 0.05). The levels of Scr, BUN, and UP in the experimental group were significantly higher than those in the control group at 24–72 hours after surgery, and the differences were statistically significant (*P* < 0.05). The Ccr and eGFR levels of patients in the experimental group were significantly lower than those in the control group at 24–72 hours after surgery, and the differences were statistically significant (*P* < 0.05).

### 3.4. Comparison of the Levels of Inflammatory Factors between the Two Groups

**Figure 9:**
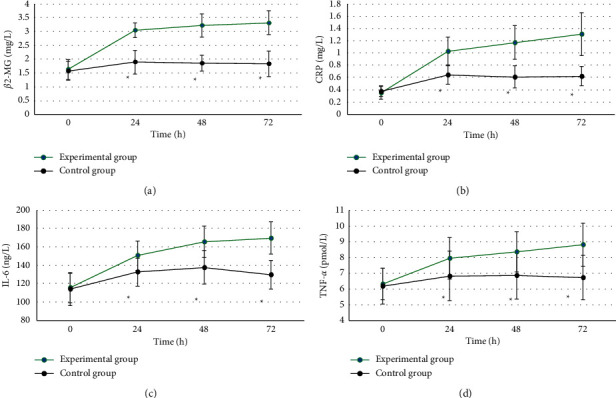
Comparison of levels of inflammatory factors between the two groups of patients (0–72 referred to “before surgery”, 24 hours, 48 hours, and 72 hours after surgery, respectively). A ∼ D showed the comparisons of *β*2-MG, CRP, IL-6, and TNF-*α*, respectively. ^*∗*^Significant difference compared with the experimental group (*P* < 0.05).

The preoperative *β*2-MG, CRP, IL-6, and TNF-*α* levels of the experimental group and the control group were not significantly different (*P* > 0.05). The levels of *β*2-MG, CRP, IL-6, and TNF-*α* in the experimental group were significantly higher than those in the control group for 24–72 hours after surgery, and the differences were statistically significant (*P* < 0.05). The specific comparison results are illustrated in Figure 9.

### 3.5. Comparison of the Incidence of CIN between the Two Groups

**Figure 10:**
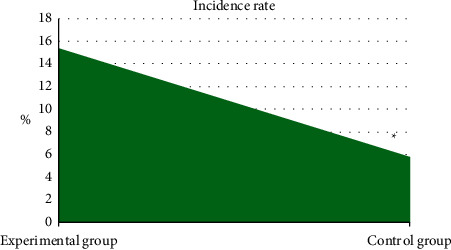
Comparison of incidence of CIN between the two groups. ^*∗*^Significant difference compared with the experimental group (*P* < 0.05).

The incidence of CIN between the two groups was compared, and the results are given in Figure 10. 8 patients in the experimental group developed CIN with an incidence rate of 15.38%, and 3 patients in the control group developed CIN with an incidence rate of 5.8%. It can be inferred that the incidence of CIN in the experimental group was significantly higher than that in the control group, and the difference was statistically significant (*P* < 0.05).

## 4. Discussion

Contrast agents play an important role in the diagnosis and interventional treatment of coronary heart disease, and the occurrence of CIN has attracted more and more attention from clinicians [21]. At present, CIN has risen to the third leading cause of iatrogenic renal failure after decreased renal blood perfusion and nephrotoxic drugs. Therefore, early evaluation and prevention of CIN are necessary [22]. In this study, 104 patients with CAHD who underwent CAG and PCI surgery within 1 week after the ECCT were treated as the research objects. They were divided into an experimental group (patients who underwent CAG and PCI within 1 week after ECCT) and the control group (patients who underwent CAG and PCI within 1–3 weeks after ECCT). All the objects were performed with the kidney CT imaging scan. In order to improve image quality, an improved image denoising algorithm based on DNST (O-DNST) was firstly proposed and compared with DSST, WT, and DNST algorithms. It was found that the RMSE and running time of the O-DNST algorithm were significantly lower than those of the DSST, WT, and DNST algorithms, and the differences were statistically significant (*P* < 0.05). This is similar to the processing performance of the original dual algorithm proposed by Foygel et al. (2016) [23] for CT image data. It shows that the O-DNST algorithm can not only effectively reduce image noise but also improve the efficiency of image processing, and has a better optimization effect on the basis of traditional algorithms. Comparison of processing effects of different algorithms revealed that the image resolution processed by the WT algorithm was still low, and that of the DSST and DNST algorithm was higher, but there were artifacts, which led to the unclear display of the subtleties of the organization. In addition, the O-DNST algorithm shows the best image clarity and tissue resolution, and the overall quality is better than other algorithms. Such results are consistent with the abovementioned quantitative results and confirmed the superiority of the algorithm in this study.

The two groups of patients were compared in terms of basic data (gender, age, height, weight, diabetes, hypertension, hyperlipidemia, mild anemia, contrast agent dose, and Mehran score), and the differences were not statistically significant (*P* > 0.05). This shows that the sample grouping of this study is reasonable and the follow-up study is feasible. Comparison on renal function indicators revealed that the levels of Scr, BUN, and UP in the experimental group were significantly higher than those of the control group 24–72 hours after surgery, while the levels of Ccr and eGFR were significantly lower than those of the control group, showing statistically significant differences (*P* < 0.05). Scr, BUN, UP, Ccr, and eGFR are all indicators used to assess renal function [24, 25], which indicates that CAG again within 1 week after ECCT may be a factor that may cause renal function damage in patients undergoing PCI. The levels of *β*2-MG, CRP, IL-6, and TNF-*α* in the experimental group were significantly higher than those in the control group for 24–72 hours after surgery, and the differences were statistically significant (*P* < 0.05). *β*2-MG, CRP, IL-6, and TNF-*α* are all sensitive markers of inflammation. The increase in the levels of these markers indicates that the body is in a proinflammatory state, indicating that CAG again within 1 week after ECCT will promote PCI The patient's serum inflammation level is increased. The incidence of CIN in the experimental group was significantly higher than that in the control group, and the difference was statistically significant (*P* < 0.05), indicating that repeated application of contrast agent in a short period of time may be a risk factor for CIN in PCI patients.

## 5. Conclusion

In this study, 104 patients with CAHD who underwent CAG and PCI surgery within 1 week after the ECCT were treated as the research objects. They were divided into an experimental group (patients who underwent CAG and PCI within 1 week after ECCT) and the control group (patients who underwent CAG and PCI within 1–3 weeks after ECCT). All the objects were performed with the kidney CT imaging scan. At the same time, the renal function indicators, inflammation indicators, and incidence of CIN were compared between the two groups of patients before and after surgery. The results showed that repeated application of the contrast agent in a short period of time can promote the increase of serum inflammation levels in PCI patients, which may be a risk factor for CIN in PCI patients. However, it was a single-center clinical randomized study with a limited sample size. In addition, none of the included subjects had kidney disease and had no significant damage to basic renal function. In addition, patients with basic kidney disease were not discussed. A large-scale randomized double-blind clinical trial of the center for the prevention and treatment of CIN would be carried out later. In conclusion, the research in this study provided theoretical support for the clinical evaluation of patients with acute renal damage caused by contrast agents.

## Data Availability

The data used to support the findings of this study are available from the corresponding author upon request.
